# Sperm DNA methylation is predominantly stable in mice offspring born after transplantation of long-term cultured spermatogonial stem cells

**DOI:** 10.1186/s13148-023-01469-x

**Published:** 2023-04-07

**Authors:** Joana B. Serrano, Nils C. Tabeling, Cindy M. de Winter-Korver, Saskia K. M. van Daalen, Ans M. M. van Pelt, Callista L. Mulder

**Affiliations:** 1 Reproductive Biology Laboratory, Center for Reproductive Medicine, Amsterdam UMC, University of Amsterdam, Meibergdreef 9, 1105 AZ Amsterdam, The Netherlands; 2 Amsterdam Reproduction and Development Research Institute, Amsterdam, The Netherlands

**Keywords:** Spermatogenesis, Spermatogonial stem cells, Spermatogonial stem cell transplantation, SSCT, Preclinical epigenetics, DNA methylation, Multi-generational mouse model

## Abstract

**Background:**

Spermatogonial stem cell transplantation (SSCT) is proposed as a fertility therapy for childhood cancer survivors. SSCT starts with cryopreserving a testicular biopsy prior to gonadotoxic treatments such as cancer treatments. When the childhood cancer survivor reaches adulthood and desires biological children, the biopsy is thawed and SSCs are propagated in vitro and subsequently auto-transplanted back into their testis. However, culturing stress during long-term propagation can result in epigenetic changes in the SSCs, such as DNA methylation alterations, and might be inherited by future generations born after SSCT. Therefore, SSCT requires a detailed preclinical epigenetic assessment of the derived offspring before this novel cell therapy is clinically implemented. With this aim, the DNA methylation status of sperm from SSCT-derived offspring, with in vitro propagated SSCs, was investigated in a multi-generational mouse model using reduced-representation bisulfite sequencing.

**Results:**

Although there were some methylation differences, they represent less than 0.5% of the total CpGs and methylated regions, in all generations. Unsupervised clustering of all samples showed no distinct grouping based on their pattern of methylation differences. After selecting the few single genes that are significantly altered in multiple generations of SSCT offspring compared to control, we validated the results with quantitative Bisulfite Sanger sequencing and RT-qPCRin various organs. Differential methylation was confirmed only for *Tal2*, being hypomethylated in sperm of SSCT offspring and presenting higher gene expression in ovaries of SSCT F1 offspring compared to control F1.

**Conclusions:**

We found no major differences in DNA methylation between SSCT-derived offspring and control, both in F1 and F2 sperm. The reassuring outcomes from our study are a prerequisite for promising translation of SSCT to the human situation.

**Supplementary Information:**

The online version contains supplementary material available at 10.1186/s13148-023-01469-x.

## Background

Spermatogonial stem cell (SSC) propagation in vitro followed by auto-transplantation (SSCT) is proposed as a future fertility treatment for men that became infertile from gonadotoxic treatments during childhood [1]. Ultimately, SSCT aims to restore spermatogenesis allowing natural conception. This therapy starts with the collection and cryopreservation of a testicular biopsy from the patient prior to gonadotoxic treatment [2, 3]. Once fertility restoration is required during adulthood, the tissue is thawed to start in vitro propagation of SSCs from the biopsy. This step is required to exponentially increase the number of stem cells that upon transplantation will colonize SSC niches and improve the success rate of the fertility treatment [1].

However, the maintenance of cells in artificial environmental conditions such as in vitro propagation throughout long periods of time could alter their epigenetic homeostasis, such as DNA methylation. In general, these culture-induced epigenetic modifications have been thoroughly studied and reproduced for multiple cell types and culture systems [4]. Therefore, it is reasonable to question if culturing stress due to long-term propagation could also result in changed DNA methylation in SSCs, which could potentially persist through the following generations, given the germline character of SSCs.

Conflicting results exist regarding potential in vitro induced epigenetic alterations in SSCs. After a 2-year period of propagation in vitro, Kanatsu-Shinohara and colleagues concluded that mouse SSCs retained the euploid karyotype and androgenetic imprint based on 5 analyzed imprinted loci (*H19*, *Meg3 IG*, *Rasgrf1*, *Igf2r* and *Peg10*) with combined bisulfite restriction analysis (COBRA) [5]. Additionally, Chip-seq analysis in mice also showed that long-term cultured SSCs preserve chromatin modifications at selected promoters as do germline progenitor cells in vivo [6]. In marmoset cultures, the DNA methylation pattern of imprinted genes *H19* and *MEST* does not change in germ cell fractions cultured for up to 21 days [7]. Conversely, Nickkholgh and colleagues investigated the epigenetic stability of human SSCs in long-term culture by analyzing the DNA methylation statuses of several imprinted regions and found that the paternally imprinted genes *H19*, *H19*-DMR (differentially methylated region), and *MEG3* were abnormally demethylated and the maternally imprinted genes *KCNQ1OT1* and *PEG3* were hypermethylated [8]. Since no genome-covering DNA methylation analysis on in vitro propagated SSCs have been performed, it is unclear how many sites are affected and whether they can persist to next generations.

Reassuringly, during development, mammals go through demethylation and remethylation to ensure the correct gene expression is maintained in the following generations [9]. This methylation reprogramming is essential to erase any epigenetic mutations that are acquired through environmental exposures during gametogenesis [10]. Any genes that managed to escape these de- and remethylation could cause epigenetic dysregulation [11], which in turn is increasingly implicated in various rare developmental syndromes and cancer, and in complex chronic diseases, such as cardiovascular disease, type 2 diabetes and obesity [12]. However, we are currently unaware whether potential culture-induced epimutations in SSCs are transferred to next generations after SSCT.

The health of SSCT-derived offspring in relation to epigenetic stability was investigated in limited studies. However, most reports are undermined by either the use of uncultured SSCs [13, 14] or resorting to additional reproductive techniques to generate offspring (i.e., in vitro fertilization (IVF), intracytoplasmic sperm injection (ICSI) or round spermatid injection (ROSI)) instead of natural conception [5, 15–17], which are thought to be prone to generate epimutations [18]. Therefore, a detailed preclinical epigenetic assessment of the offspring derived from male parents that were transplanted with in vitro propagated SSCs in a model organism is warranted before clinical SSCT application.

Here, we evaluate the molecular epigenetic stability of SSCT-derived offspring by performing an in-depth DNA methylation analysis on sperm derived from the first and second generation of SSCT-derived mouse offspring and compare this with that of control sperm.

## Results

**Fig. 1:**
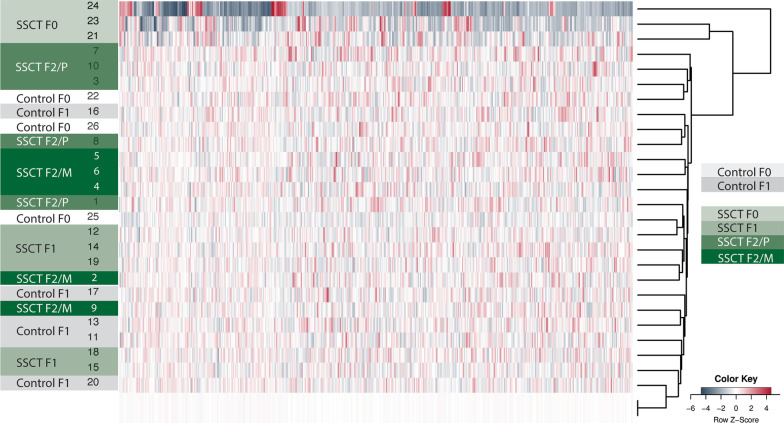
Global unsupervised clustering heatmap of the average methylation of all the samples. The heatmap clustering of all the samples is based on the significantly differential methylation between each sample (*n* = 3 in the F0, *n* = 5 per offspring subgroup in the F1 and F2) and the average methylation of all the samples (in duplo), included in the heatmap as the last 2 rows in medium light gray for the top-500 most altered DMRs (both hypermethylated and hypomethylated)

In this study, we investigated whether the DNA methylation status is different between sperm from SSCT-derived male offspring (F1 and F2) and control male offspring at three months of age using reduced-representation bisulfite sequencing (RRBS). First, we performed unsupervised clustering of differentially methylated regions (DMRs) in all samples (Fig. 1). As expected, due to age difference and different background environment, SSCT F0 sperm samples from the transplanted animals clustered away from the offspring sperm samples control F1 and SSCT F1 and SSCT F2. For all offspring, no distinct arrangement of any of the groups could be identified.

Statistical examination of the RRBS DNA methylation datasets revealed that sperm derived from the transplanted male (F0) showed 3.63% significant differences among all measured CpGs and 5.63% in DMRs, compared to control F0. In F1 and F2 sperm, less than 0.4% of all measured differentially methylated CpGs (DMCs) and DMRs showed significant methylation differences (≥ 25%) between SSCT and F1 control animals (Table 1).

**Table 1:** All DMCs and DMRs that are significantly different between control F0 or control F1 and various SSCT groups and subgroups (*n* = 3 in the F0, *n* = 5 per offspring subgroup in the F1 and F2)

	All measured DMCs	Number of CpGs displaying > 25% difference	Percentage of > 25% over of all measured CpGs
SSCT F0	82,296	2984	3.63%
SSCT F1	714,806	2892	0.40%
SSCT F2	504,893	833	0.16%
SSCT F2/M	609,442	2695	0.44%
SSCT F2/P	664,355	2823	0.42%

	All measured DMRs	Number of DMRs displaying > 25% difference	Percentage > 25% difference of all measured DMRs
SSCT F0	19,621	1105	5.63%
SSCT F1	138,480	500	0.36%
SSCT F2	113,616	153	0.13%
SSCT F2/M	128,075	477	0.37%
SSCT F2/P	133,762	491	0.37%

F2/M—F2 generated from the maternal line, F2/P—F2 generated from the paternal line

**Fig. 2:**
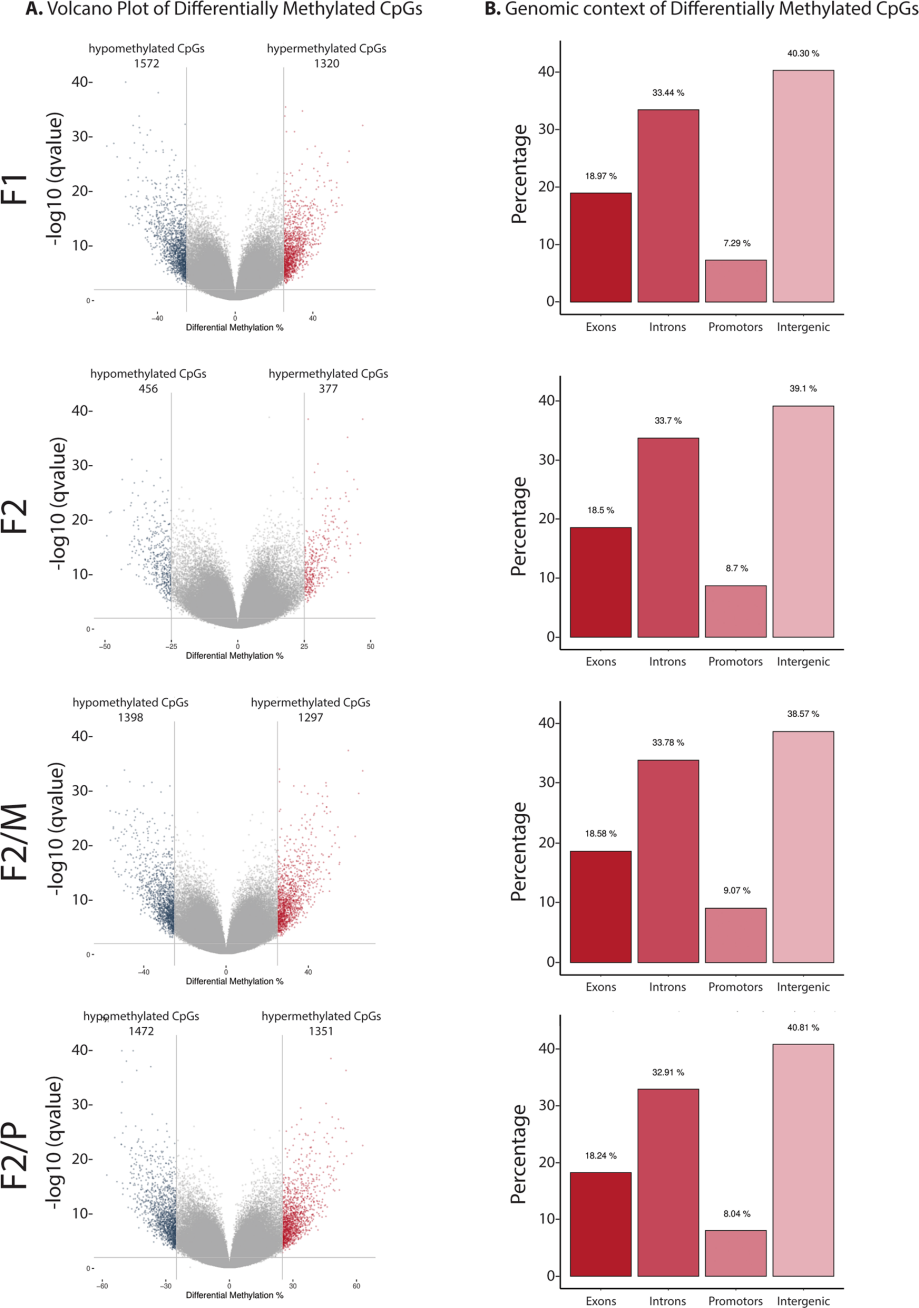
Differential analysis for CpG sites of the SSCT F1 and F2 compared to control F1. **A** Volcano plot shows the number of CpGs with changed patterns of CpG methylation between SSCT samples and control samples significantly higher or lower than the 25% difference cut-off and considering a *q*-value threshold of 0.01. Values on the *x*- and *y*-axes are percent methylation differences and negative log10 of the corrected *p*-values, respectively. **B** The distribution of differentially methylated CpGs (DMCs) over DNA regions exons, introns, promoters and intergenic regions per generation. F2/M—F2 generated from the maternal line, F2/P—F2 generated from the paternal line

**Fig. 3:**
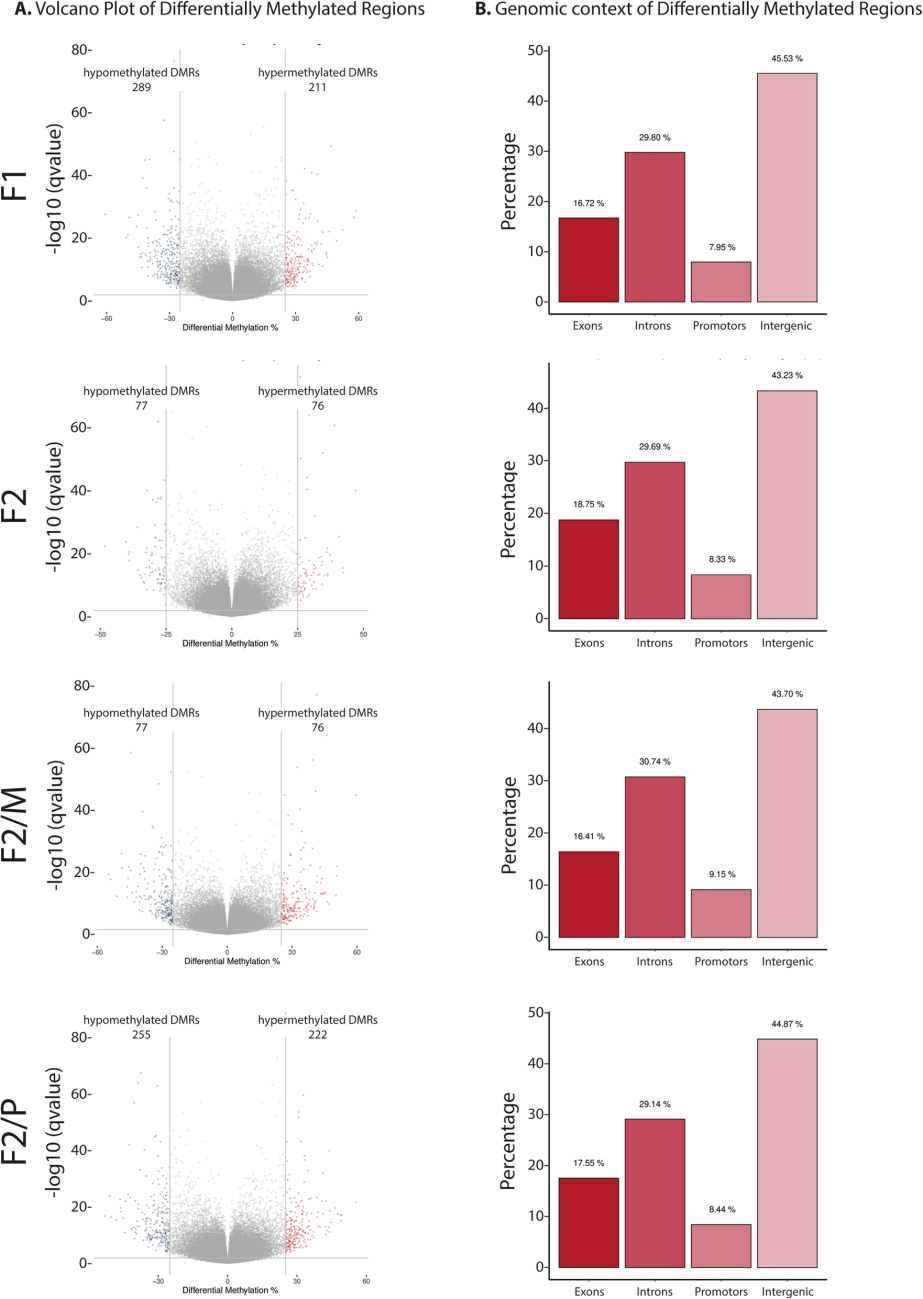
Differential analysis for DMRs of the SSCT F1 and F2 offspring compared to control F1. **A** Volcano plot shows the number of DMRs with changed patterns of methylation between SSCT samples and control samples significantly higher or lower than the 25% difference cut-off and considering a *q*-value threshold of 0.01. Values on the *x*- and *y*-axes are percent methylation differences and negative log10 of the corrected *p*-values, respectively. **B** The distribution of differentially methylated regions (DMRs) over DNA regions exons, introns, promoters and intergenic regions. F2/M—F2 generated from the maternal line, F2/P—F2 generated from the paternal line

DMCs and DMRs with altered patterns of methylation > 25% between SSCT F1 and control samples are visually represented in Volcano plots (Figs. 2A, 3A, respectively).

Subsequently, we assessed the annotated genomic region of the DMCs and DMRs categorized by exons, introns, promoters and intergenic regions. We found that DMCs and DMRs were similarly distributed over the various genomic regions and the different groups (Figs. 2B, 3B, respectively) with approximately 16–19% of the DMCs and DMRs annotated to exons and 7–9% to promoters.

As intergenic regions represent the most affected DMRs in these datasets (Fig. 3B), DMRs that displayed differential methylation spanning from F0 to F2 were analyzed against datasets for known sequences of interest such as enhancers (http://www.enhanceratlas.org/downloadv2.php, sperm) [19] or intracisternal A-particle (IAP) sequences (multiple organs) [20, 21] that have been previously characterized in mice and could impact the health of SSCT in multiple generations. We did not see any DMRs in enhancers (Additional file 1: Table S1). Although we do see DMRs in the entire intergenic regions 4459, 6440 and 7407 on chromosome 3, 4 and 5, respectively, that also contain IAPs IAPLTR2a-ERVK-LTR and IAPLTR1_Mm-ERVK-LTR (Additional file 1: Table S1), there was no overlap of these IAP elements with that of the DMRs within these intergenic regions (Additional file 1: Fig. S1).

Next, we performed gene ontology analysis based on the identified DMRs and DMCs. For both exon and promoter regions, no biological association of genes annotated to DMRs could be identified in the F1 offspring. However, gene ontology analysis suggested some differences in multiple biological processes in DMCs annotated to exons in SSCT F1, while both DMCs and DMRs in SSCT F2 offspring presented pathways affected in various biological processes compared to control (Additional file 1: Fig. S2). Methylation differences between control F1 and SSCT F1 in CpGs in exons were among others most prominently associated with cyclic nucleotide catabolic process (e.g., *Cnp*, *Pde4b*, *Pde4a*, *Pde5a*, *Pde2a*). While only one pathway was significantly affected in SSCT F2 exons compared to control F1, namely ‘negative regulation by host of viral transcription’ (e.g., *Ccl3*, *Tfap4*), and in promoters the most affected pathways were associated with biological processes such as ‘orbitofrontal cortex development,’ ‘cerebral cortex tangential migration using cell–cell interactions,’ ‘substrate-dependent cerebral cortex tangential migration’ and ‘postnatal olfactory bulb interneuron migration’ (e.g., *Fgfr1*, Additional file 1: Fig. S2). When comparing SSCT F2/M with control F1, the DMCs in exons related to ‘memory’ (e.g., *Mapt*, *Slc6a4*, *Rin1*) and ‘regulation of chemotaxis’ (e.g., *Sema4f*, *Ccl3*, *Sema3g*), while promoters were predominantly associated with ‘tunicamycin response’ (e.g., *Clu*, *App*), ‘cysteine meta- and catabolism’ (e.g., *Csad*, *Agxt*) and ‘memory T cell activation’ (e.g., *Tcirg1*, *Tnfsf4*). Finally, only exons presented DNA methylation differences in SSCT F2/P compared to control F1, with the most affected DMCs associated with regulation of ‘signaling receptor activity’ (e.g., *Dapk1*, *Lypd1*, *Fbxw7*), ‘axon guidance’ and ‘neuron projection guidance’ (e.g., *Gata3*, *Etv4*, *Mypn*), and the altered DMRs in biological processes such as ‘glutamine and glutamate processes’ (e.g., *Gls*, *Gls2*). These results again suggest no common biological process for all generations. We also examined if any of the affected DMRs and DMCs were present in any known parentally imprinted genes in mice, but imprinting appeared unaffected in all generations.

To confirm the aberrant DNA methylation profiles of ≥ 25% differences in methylation found with RRBS in sperm, a validation of genetic regions annotated to exons and promoters was performed using high-resolution bisulfite Sanger PCR sequencing (BSP). Throughout generations (F0, F1 or F2), 30 genes with significantly hyper- or hypomethylated DMRs were identified (Table 2) in sperm from SSCT compared to control. From these, genes were selected if they appeared altered in more than one generation (Table 2), revealing eight genes. None of these genes were spanning in all three generations (F0, F1, F2). The SSCT F0 vs control F0 was not included in the validation experiments because DBA2/J SSCT-derived donor sperm was generated in a different background mouse strain (WBB6F1/J W/W-v) somatic niche, harvested at an older age (18 months), and the yield of DNA was low.

**Table 2:** Shared genes in bold with altered DMRs between generations SSCT F0, SSCT F1 and SSCT F2 in exon and promoter regions that were analyzed for selection for further validation experiments

Overlap	Genes	SSCT F0 + SSCT F1 + SSCT F2/M	SSCT F0 + SSCT F1 + SSCT F2/P	SSCT F0 + SSCT F1	SSCT F1 + SSCT F2	SSCT F1 + SSCT F2/M	SSCT F1 + SSCT F2/P
Promoter	***Zfp229***	−	−	−	+	+	+
	***F930017D23Rik***	−	−	−	+	−	+
	*Bag2*	−	−	−	−	−	+
	***Nudt1***	−	−	−	−	−	+
	*Mrm2*	−	−	−	−	−	+
	*Gm12011*	−	−	−	−	−	+
	*Slc13a4*	−	−	+	−	−	−
	*Zfp513*	−	−	−	−	+	−
Exon	***Gm6590***	−	−	−	+	+	+
	***F930017D23Rik***	−	−	−	+	−	+
	***Gpx8***	+	−	+	−	+	−
	***Tal2***	+	−	+	−	+	−
	***C130012C08Rik***	−	+	+	−	−	+
	*BC035947*	−	−	+	−	−	−
	*Mars*	−	−	+	−	−	−
	*Tet1*	−	−	+	−	−	−
	*Slc13a4*	−	−	+	−	−	−
	*Capn11*	−	−	+	−	−	−
	*Pou2f2*	−	−	+	−	−	−
	*Dlg4*	−	−	−	−	+	−
	*Ppm1g*	−	−	−	−	+	−
	*Arfgef3*	−	−	−	−	+	−
	*Kctd10*	−	−	−	−	+	−
	*Myo1h*	−	−	−	−	+	−
	*Gm15966*	−	−	−	−	+	−
	*Mt3*	−	−	−	−	−	+
	*Epha2*	−	−	−	−	−	+
	*Kcnk10*	−	−	−	−	−	+
	*Nudt1*	−	−	−	−	−	+
	*Gm12011*	−	−	−	−	−	+
	*Elmsan1*	−	−	−	−	−	+
	*Tmprss9*	−	−	−	−	−	+

F2/M—F2 generated from the maternal line, F2/P—F2 generated from the paternal line

**Fig. 4:**
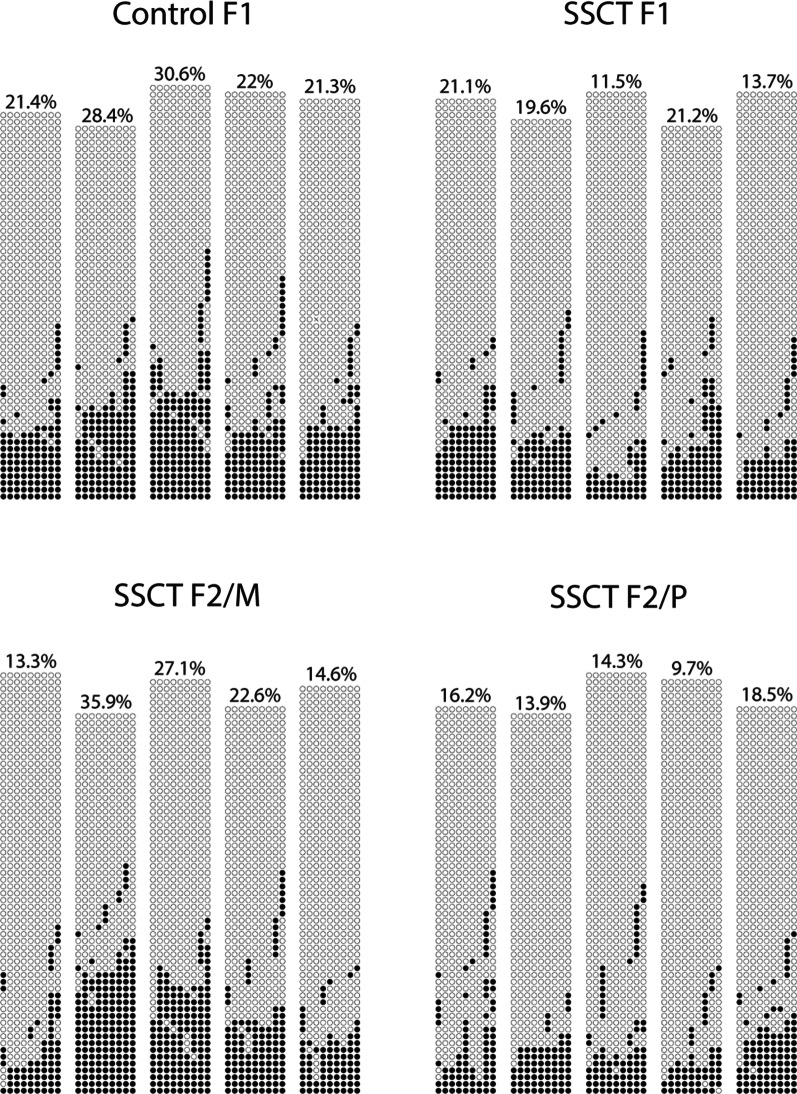
Validation of RRBS results with BSP of *Tal2* from individual sperm samples. Overall CpGs presented the following averages per group: control F1 24.7% ± 4.4%, SSCT F1 17.4% ± 4.5%, SSCT F2/M 22.7% ± 9.3%, SSCT F2/P 14.5% ± 3.2% presenting the highest difference of 10% methylation difference between SSCT and control (*n* = 5 per offspring subgroup in the F1 and F2). F2/M—F2 generated from the maternal line, F2/P—F2 generated from the paternal line

Validation by BSP of the selected statistically significant ≥ 25% hypo- and hypermethylated genes found by RRBS in both F1 and F2 generations could not be confirmed (Additional file 1: Table S3). However, for one gene designated *Tal2*, a 10% methylation difference in several CpGs could be observed (Fig. 4), with a decreased DNA methylation level in SSCT F1 mice sperm compared to control F1 mice sperm, thereby confirming RRBS results with a lower percentage of methylation difference.

**Fig. 5:**
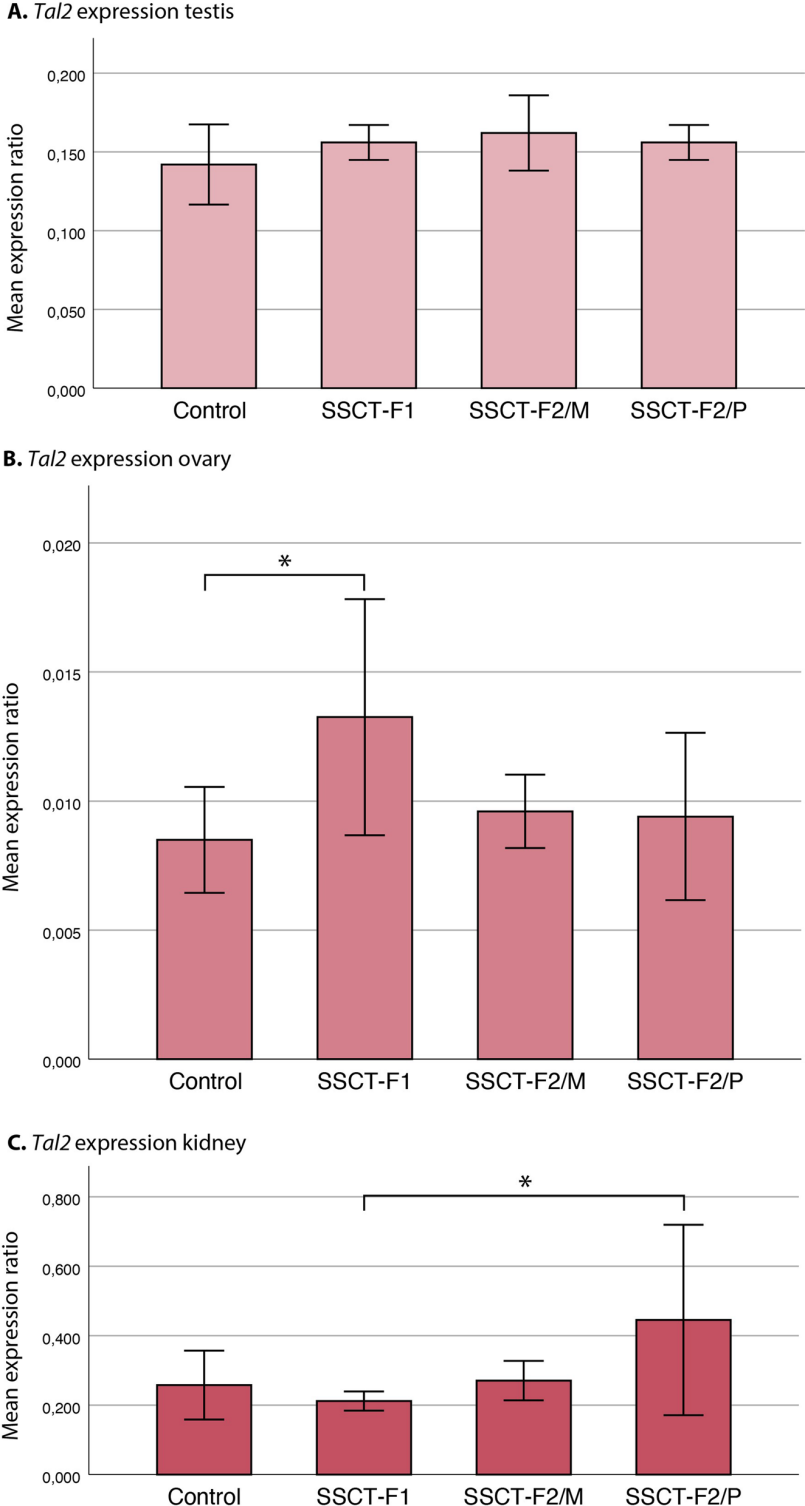
Comparative *Tal2* gene expression analysis with RT-qPCR. **A** testis, **B** ovary, and **C** kidney between control F1 and SSCT offspring in F1 and F2/M and F2/P. The bar chart and statistical analyses with ANOVA followed by Tukey’s post hoc testing represent the RNA expression between groups of all generations, (*n* = 5 per organ, per offspring subgroup in the F1 and F2, RT-PCR performed in technical triplos). *Statistically significant differences observed between groups (*p* < 0.05). F2/M—F2 generated from the maternal line, F2/P—F2 generated from the paternal line

To determine if the epigenetic variations found in *Tal2* in RRBS and BSP analysis led to altered gene expression, we quantified the RNA levels of *Tal2* in organs that normally express this gene (namely, testis [22, 23], ovary and kidney, Search: Tal2—The Human Protein Atlas) with RT-qPCR to explore the potential biological impact. Gene expression analysis found no statistically significant differences between control F1 and SSCT F1, SSCT F2/M or SSCT F2/P groups in *Tal2* expression in testis biopsy (Fig. 5A). However, there is a statistically significant difference between groups in *Tal2* expression for ovary (Fig. 5 B, *p* = 0.029). Post hoc analysis determined this significance originates from the difference between SSCT F1 and control F1. A higher expression in SSCT F1 is consistent with the lower methylation levels as found in BSP analyses in sperm samples. Statistically significant differences were also found between groups in *Tal2* expression for kidneys (Fig. 5C, p = 0.036). This significance originates from the differences between SSCT F1 and the SSCT F2/P group; however, expression did not differ compared to control F1 animals for all SSCT generations.

## Discussion

SSCT, as a novel stem cell therapy, aims to permanently restore fertility in men after gonadotoxic treatments during childhood. Potential environmental stress during long-term SSC propagation, required for successful transplantation, could affect early epigenetic events during spermatogenesis, which in turn can affect the health of the offspring born from these cells.

In this study, we performed RRBS to study DNA methylation in a multi-generational mouse model after transplantation of long-term cultured SSCs into infertile F0 fathers. We found no major differences in methylation between SSCT-derived offspring compared to control, both in sperm of the F1 and F2 generation. This suggests that the stability of DNA methylation is preserved or restored during development after SSCT. These results are in agreement with a detailed life-long health assessment of the SSCT-derived mice and their second generations that SSCT offspring have similar health conditions during childhood and adulthood compared to control [24].

The strength of our study is the use of a broad genome methylation analysis such as the RRBS technique, which warrants a non-biased approach of studying potentially affected DNA methylation throughout the genome in areas enriched in CpGs. Therefore, this study investigated for the first time the epigenetic effects in multiple generations of SSCT-derived offspring from long-term cultured SSCs.

The weakness of our study is that after arranging all the samples through unsupervised clustering indicated that sperm from transplanted males (F0) differed from all other samples. The differences found in SSCT F0 compared to control F0 may be attributed to the persistence of culture-induced epimutations in SSCs after SSCT-derived spermatogenesis but are not transferred to the following generations. However, it might also well be that the distinct grouping of SSCT F0 fathers originates from the difference in age of the animal at sperm collection (18 months F0 versus 3 months F1 and F2) [25] or the fact that DBA2/J donor SSCs were transplanted in a different mouse strain background (W/W-v in WBB6F1/J strain) somatic niche, which may differ from a DBA2/J somatic environment and therefore represents a weakness of our study. Still, these differences do not persist in subsequent generations, and it can therefore be assumed that the majority of epimutations are reset during spermatogenesis and/or embryogenesis in the F1. In addition, no distinct grouping based on their pattern of methylation differences could be detected in SSCT F1 and SSCT F2, compared to control F1, indicating that the individual samples in SSCT F1 and SSCT F2 have variable epigenetic patterns indistinguishable from each other in all offspring and no specific pattern to a certain generation. By selecting sperm as a sample, we benefited from the homogeneity of the sperm cell pool, instead of using a heterogeneous somatic tissue sample that could mask any alterations in the DNA methylation, due to the noise of the multiple and diverse cell types. However, DNA methylation differences in the F0 sperm might cause abnormalities in F1 somatic tissues of the F1. Reassuringly, from a previous study we determined that if there were any alterations, they are not contributing in a major way to the offspring health [24].

After statistical analysis of all RRBS methylation differences in SSCT F1 and SSCT F2, less than 0.5% of the DMCs and DMRs were found to be statistically significant hyper or hypomethylated with ≥ 25% difference. After genomic annotation of the methylation differences, only a minority of the DMCs and DMRs were annotated to exons and promoters. Given the location of these modifications, they could possibly result in biologically relevant modifications that have pathological consequences. However, focusing on these two genomic regions, BSP could not confirm the > 25% hyper and hypomethylation for most genes that were hyper or hypomethylated in multiple generations according to RRBS. Only the hypomethylation in *Tal2* was verified, although at a lower difference (~ 10%).

To investigate whether the hypomethylation of *Tal2* could incur translational differences in the SSCT offspring, gene expression analyses were performed, and no differences were found on *Tal2* expression in the testis of SSCT offspring compared to control. However, the expression results may be biased, as sperm cells account for a small part of the total testis tissue. In the ovary, where *Tal2* is also expressed, the lower methylation led to a significantly increased gene expression in SSCT F1 compared to control F1, which did not persist to the F2 generation either via the female or male origin. Conversely, a decreased, rather than increased, *Tal2* expression is generally found in epithelial ovarian carcinoma [26, 27]. It needs to be noted that a previous multi-generational study [24] investigating the general health of the offspring after SSCT with long-term cultured SSCs, found three out of forty-seven cases of sarcoma specifically in the female reproductive system (ovary, uterus) in SSCT F1 and none in control. Whether this specific malignancy found in the previous study is associated with a higher expression of *Tal2* as found in this study is still unknown, since sarcomas have a divergent molecular pathology compared to carcinomas. On the contrary, while the left ovary weight was the same for all groups, the right ovary actually had a significantly lower weight in SSCT F1 (at 18 months of age) compared to control, despite all the females being fertile [24]. A lower ovary weight is reassuring since it indicates that these animals probably do not have cancer, as malignancies would be associated with increased weight. More research is necessary to investigate this potential association between *Tal2* expression and ovarian diseases. Therefore, ovarian malignancies should be monitored closely in offspring of future SSCT-derived girls and women. No differences in weight or histopathology were found in both kidneys of SSCT offspring compared to controls, also indicating no functional alteration of the hypomethylation of *Tal2* in this organ.

In our results, we did not find any alterations in parentally imprinted genes. Our results are in line with the outcome from Goossens et al*.*, who analyzed the health of the offspring as well as DNA methylation patterns of *α-Actin* and imprinted genes *Igf2* and *Peg1*, which were not different among controls and first and second-generation offspring after SSCT using uncultured SSCs. This corroborates the finding that murine SSCs after long-term culture are epigenetically stable based on a few imprinted loci [5]. In addition, even though the majority of DMRs were present in intergenic regions, no intergenic DMRs spanning all generations (F0-F2) contained known enhancers and IAP elements. The latter have been correlated to transgenerational inheritance of epigenetic traits [28]; therefore, it is reassuring that these elements are stable in SSCT offspring. Our results are consistent with our previous results in a larger cohort of animals, where we found that physical and developmental parameters in the SSCT offspring are similar to control [24]. However, since human SSCs do show epigenetic differences after culture [29, 30], epigenetic studies are needed during the follow-up of the children in Phase I clinical trials of SSCT. Before clinical implementation of SSCT as treatment in human, some safety features of the procedure still must be investigated [31, 32]. The optimal cryopreservation protocol of testicular tissue has been established but has not been standardized yet [32]. As the methods for SSC propagation are optimized in rodents, the human culture still requires fine tuning to achieve the same results. Therefore, as new culturing conditions (temperature, growth factors, supplements) are advanced in the human culturing method, their effects should also be studied in human both preclinically and in Phase 1 clinical trials.

## Conclusions

We can conclude that some epimutations exist in sperm from SSCT-derived offspring but reassuringly these do not seem to have major pathological effects. This study on epigenetic stability and the previous study on general health in SSCT offspring in multi-generations can be used to request ethical approval for the introduction of SSCT in phase 1 clinical trials, along with a follow-up of the children, once human SSC culture is optimized and confirmed epigenetically safe.

## Methods

### Study design

**Fig. 6:**
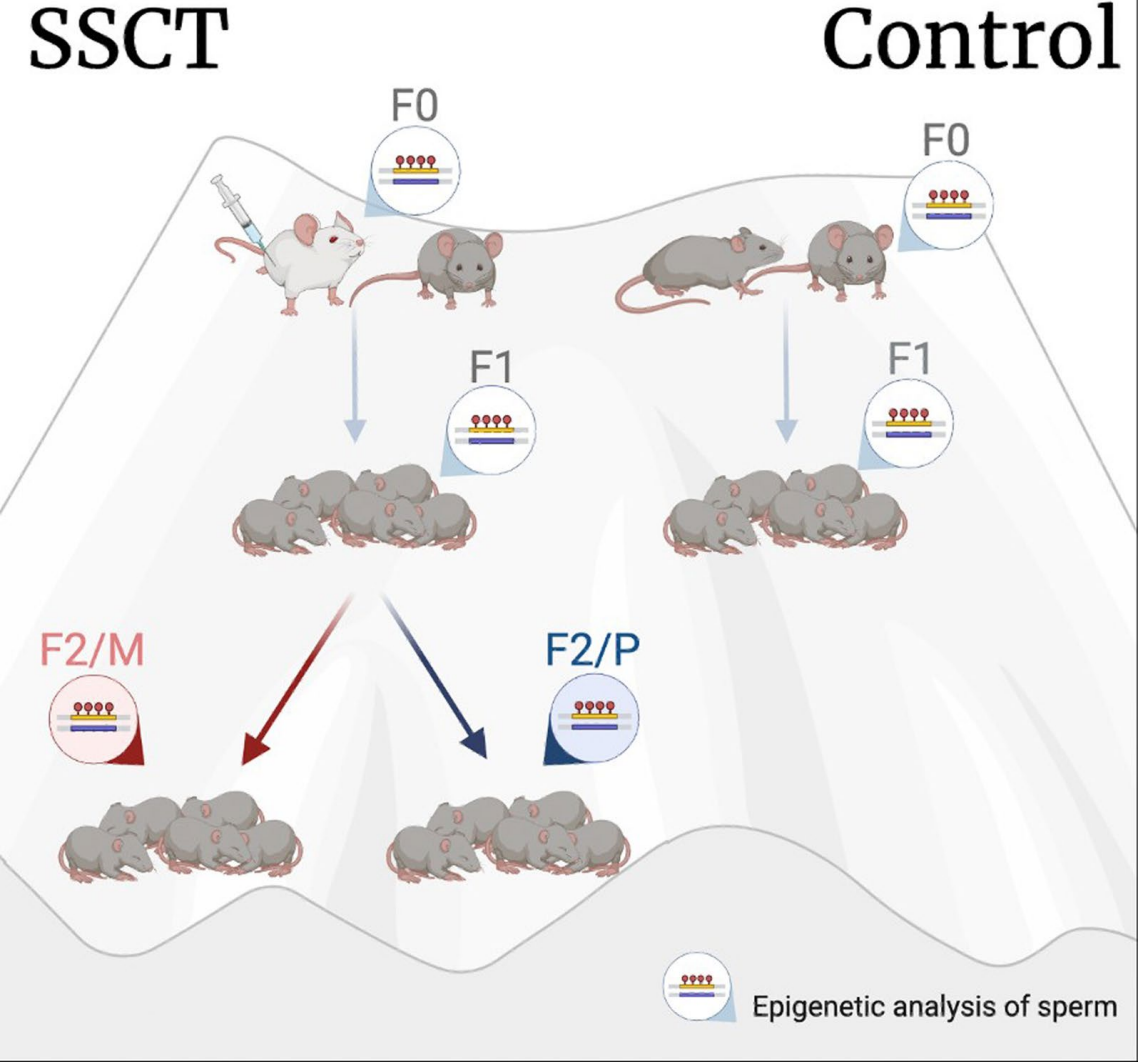
Multi-generational mouse model of SSCT offspring generation. F0—transplanted mice, F1—first generational born after SSCT, F2—second generational born from one SCCT-derived parent F2/M—F2 generated from the maternal line, F2/P—F2 generated from the paternal line. Sperm was collected from five males of each generated offspring for comparison of DNA methylation status (*n* = 3 in the F0, *n* = 5 per offspring subgroup in the F1 and F2)

Mouse neonatal SSCs (4–8 days postpartum, DBA/2 J) were propagated in vitro and subsequently transplanted into sterile males (W/W-v) as part of our previous study on the general health of SSCT-derived offspring [24]. SSCs were cultured as described previously [15, 24, 33]. In brief, the testis biopsies were digested in collagenase, followed by Trypsin/EDTA, HBSS and DNase. The cells were plated in 0.1% gelatin coated plates at a density of 200.000 cells/well in supplemented Stem Pro-34. Floating cells were passaged to non-coated plates. After the third passage, cells were cultivated with mouse embryonic fibroblasts. Ultimately, seven different cell lines cells were cultured up to 50 days to at least passage 5 before transplantation. The offspring were generated by mating control males (DBA/2 J, *n* = 3) and transplanted male mice (sterile W/W-v transplanted with DBA2/J SSCs, *n* = 3) with control females (DBA/2 J, *n* = 28). In the control group, only one generation was bred (inbred DBA/2 J) for welfare reasons according to the reduction principle of the 3Rs, while in the SSCT group two generations were bred, a first generation derived from transplanted male and wildtype female mice (SSCT F1). A second generation was bred by mating control males with SSCT F1 females as mothers (generating from the maternal line, SSCT F2/M) or by mating control females with the SSCT F1 males as fathers (generating from the paternal line, SSCT F2/P) (Fig. 6).

### Mice husbandry and welfare

The experiments were performed according to the European Directive 2010/63/EU on the protection of animals used for scientific purposes and licensed by the Dutch competent authority (AVD1180020171524). The Animal Welfare Body of the Amsterdam UMC, location the Academic Medical Center, was responsible for overseeing the experiments. The animals were in a 14:10 h reversed day/night cycle, with a standard rodent chow diet (Envigo 2016 Teklad global 16% protein rodent diet) and water available ad libitum in open cages. The mice were housed socially with their littermates of the same sex, and the cages were switched frequently throughout the room. The animal caretakers and researchers were blinded for the whole duration of the study. Daily observations were recorded in a logbook that registers the handling of each animal. When required, a veterinarian or animal welfare officer was contacted for advice on welfare issues. An independent researcher was responsible for administrative monitoring throughout the study: arranging breeding schemes; animal selection for this specific study and breeding and kept the key for the blinded researchers. When choosing animals for the breeding schemes, the use of animals couples derived from the same parents was avoided to circumvent parental effects.

### Biological material collection

Animals were sacrificed at 3 months of age by C02 asphyxiation according to the guidelines. Epididymal sperm was isolated from control F1, SSCT F1, SSCT F2/M and SSCT F2/P offspring (*n* = 5 for each). For the three SSCT F0 transplanted W/W-v males and DBA2/J controls, donor-derived DBA2/J and control epididymal sperm were isolated at 16–18 months of age, as they were needed to breed the offspring for a prolonged time. In addition, the following organs were harvested from the F1 and F2 mice at 3 months of age and stored at − 80 °C: testis, ovaries, uterus, bladder, intestine, spleen, stomach, liver, kidneys, lungs, heart, thymus and brain for validation studies. In all groups, sperm was isolated from the cauda epididymis in a droplet of human tubular fluid (HTF) medium by piercing the tissue with fine tweezers and a needle, followed by incubation for 1 min at 37 °C degrees. The presence of sperm was confirmed under the microscope. After removing the tissue, the HTF with sperm was collected in an Eppendorf and washed in 500uL phosphate-buffered saline (PBS). For purification of the sperm, the suspension was carefully pipetted into a falcon with 2 mL PureSperm 70% (Nidacon) while having the falcon almost horizontal and centrifuged for 20 min at 300 RCF no break at room temperature (RT). The pellet was then washed with 5 mL HBSS or PBS and centrifuge for 10 min at 500 RCF after which the pellet was SNAP frozen and stored at − 80 °C for further analysis.

### DNA isolation

DNA was extracted from the pelleted sperm by resuspending in 250 µl of sperm lysis buffer (0.075 M NaCl; 0.025 EDTA; 0.0275 M NaOH; 10% SDS, pH 8.0), transferred to a 1.5 ml tube together with 25 µl proteinase K (20 mg/ml) and 2.5 µl 1 M DTT. The tube was vortexed and placed overnight at 55 °C in a shaking heat block at 750 rpm. Subsequently, 92.5 µl of 6 M NaCl was added, followed by centrifugation for 5 min at full speed in a microcentrifuge. The clear supernatant was transferred to a new tube and centrifuged again for 5 min at full speed. Glycogen solution (20 mg/ml) was added to a final concentration of 0.1 µg/µl along with 0.7 volume of isopropanol and mixed gently and incubate for 1 h at − 20 °C and centrifuged for 15 min at full speed at 4 °C. The pellet was washed with 70% EtOH and air dried for 5–10 min and dissolved in 20 µl Tris EDTA buffer (TE) by pipetting up and down. The concentration of the isolated DNA was measured with a Nanodrop.

### Bisulfite conversion and RRBS

The bisulfite conversion and reduced-representation bisulfite sequencing (RRBS) were performed at Diagenode (Belgium). Briefly, the DNA concentration of the sperm samples was measured using the Qubit® dsDNA BR/HS Assay Kit (Thermo Fisher Scientific). DNA quality of the samples was assessed with the Fragment Analyzer™ and the DNF-487 Standard Sensitivity or the DNF488 High Sensitivity genomic DNA Analysis Kit (Agilent). RRBS libraries were prepared using the Premium Reduced-Representation Bisulfite Sequencing (RRBS) Kit (Diagenode Cat# C02030033). Following library preparation, samples were pooled (pools of eight or nine samples). After the final library amplification, the PCR clean-up was completed using 1.45 × beads/sample ratio of Agencourt® AMPure® XP (Beckman Coulter). RRBS library quality control DNA concentration of the pools was measured using the Qubit® dsDNA HS Assay Kit (Thermo Fisher Scientific). High Sensitivity DNA chip for 2100 Bioanalyzer (Agilent) was used to check the profile of the pools. In case of too high adapter dimer peaks, the pools were size selected one more time using 1.45 × beads/sample ratio of Agencourt® AMPure® XP (Beckman Coulter) and quality control steps were performed again. HiSeq3000 (Illumina) was used to sequence the RRBS library pools using 50 bp single-read sequencing (SR50). Bisulfite conversion efficiency was higher than 99% for all samples.

### Gene ontology analyses

Gene ontology analyses and graphical representation of gene enrichment were performed using ShinyGO enrichment tool (Accessed 25th July, 2022 at http://bioinformatics.sdstate.edu/go/) [34].

### Quantitative Bisulfite Sanger sequencing PCR

For validating RRBS results, bisulfite sequencing PCR (BSP) was performed as previously described [8]. Briefly, bisulfite conversion was performed with EZ DNA Methylation-Gold Kit from Zymo research according to the instructions on the kit. Primers were designed using Methprimer 2.0 [35] for the sequence of interest. Two sets of primers that covered the highest number of CpGs per gene were selected for amplification of bisulfite-converted DNA for the specific region of interest. Primers were validated on forehand and optimized using control sperm DNA (C57BL6/J) and a commercially available unmethylated control (EpigenDx) (Additional file 1: Table S2). Ultimately, primers were chosen depending on the successfulness of the PCR results (single bright band after gel electrophoresis), and the highest number of CpGs that the primers covered within the region of interest.

Amplification was performed with the Epitec Master Mix PCR product or AmpliGold Polymerase PCR product. Either the PCR product was used directly for molecular cloning, or after clean-up with Roche’s High Pure PCR Cleanup Kit according to the manufacturer’s description. For cloning, the DNA was intergraded into the TOPO vector using the TOPO TA Cloning kit by Invitrogen to allow sequencing from the T7 site according to the manufacturer’s protocol. After cloning in NEB 5-α competent E. coli cells, these bacteria were grown overnight on an agar plate with 50 μg/ml ampicillin, 100 mg/ml Isopropyl β-d-1-thiogalactopyranoside (IPTG) and 100 mg/ml of 5-bromo-4-chloro-3-indolyl-β-D-galactopyranoside (X-GAL). Overall, 60 clones per gene from all 20 samples (*n* = 5 for control F1 and SSCT F1, SSCT F2/M and SSCT F2/P) were Sanger sequenced with Big Dye Buffer, Big Dye Terminator and T7 primers. The sequencing quality was checked in Codon Code (https://www.codoncode.com/). The sequences were finally analyzed for their CpG methylation status using the web-based bisulfite sequencing analysis tool QUMA and pairwise analyses were performed to align bisulfite sequences with their genomic sequence (http://quma.cdb.riken.jp/). Ultimately, graphical representations of the bisulfite alignment between the target genomic sequence and each bisulfite sequence were displayed as DNA methylation patterns in black/white circles and downloaded from QUMA.

### Gene expression analysis

RNA was isolated from selected organs using RNeasy Fibrous Tissue Mini Kit (Qiagen). DNA was removed with the DNAse Max kit (Qiagen). RNA concentration was measured with Nanodrop, and its integrity was verified on the 2100 Bioanalyzer (Agilent Technologies). RNA samples were cleaned with the Qiagen Mini Elute kit when the ratio 260/230 was too low. cDNA was synthesized using random primers (50 ng per 1 ug RNA, Promega), 1.25 µl of 10 mM dNTP-mix (Promega) and M-MLV Reverse Transcriptase (Invitrogen). For qPCR, oligonucleotide primers were designed using the NCBI website and optimized for the target gene *Tal2* (Additional file 1: Table S2), and three reference genes *Sart3*, *Oraov1*, *Fam192*, which were tested and selected for stable expression for all used tissues and cells (Additional file 1: Table S2). qPCR reactions were carried out in triplicate with the LightCycler 480 SYBR Green I Master (Roche), 0.5 μM of forward and reverse primers, and a final volume of 20 μl in 96-well plates using the LightCycler480 PCR machine (Roche). The LinReg software (version 11.0, http://LinRegPCR.nl) [36] was used according to the manufacturer's instructions to calculate the starting concentration (N0 value) of the gene transcripts based on the fluorescence level per cycle number in each sample. PCR specificity of the amplified products was checked on a 3% agarose TBE gel with ethidium bromide and visualized on a Gel Doc XR system (Bio-Rad) to confirm the presence of a single band at the correct size.

### Statistical analysis

For sperm collection, five male mice per generation (sub)group were selected at random using an online random number generator but avoiding brothers or sisters in the same group. The DNA methylation levels between groups were compared as follows: control F0 vs SSCT F0, control F1 compared to SSCT F1, SSCT F2, SSCT F2/M and SSCT F2/P.

Differential methylation analysis to investigate the methylation status of CpG-rich regions of the genome was performed using the Methylkit v1.7.0 [6], a R/Bioconductor package. All CpGs results were filtered for good coverage (CpGs with coverage less than 10X in all samples per comparative group were discarded, while CpGs with coverage higher than the 99.9th percentile were also discarded). The differentially methylated regions (DMRs), with a window and step size of 1000 bps, were subsequently annotated based on their CpG context or genomic region. Differentially methylated CpGs (DMCs) and DMRs were calculated and a > 25% change of methylation between SSCT and control samples with a *q*-value < 0.01 was considered statistically significant and potentially biologically relevant [37, 38]. For gene expression analyses with RT-qPCR, the mean expression ratios were statistically evaluated using IBM SPSS Statistics 28 by ANOVA followed by a Tukey post hoc analysis.

## Supplementary Information

**Additional file 1.** Supplementary tables and figures.

## Data Availability

All data is included in this published article and its supplementary information files. The datasets generated and/or analyzed during the current study are available in the GEO repository (number GSE213399).

## Abbreviations

SSCT: Spermatogonial stem cell transplantation
SSC: Spermatogonial stem cell
COBRA: Combined bisulfite restriction analysis
*H19*: H19 Imprinted Maternally Expressed Transcript
*MEST*: Mesoderm specific transcript
DMR: Differentially methylated region
*MEG3*: Maternally Expressed 3
*KCNQ1OT1*: KCNQ1 Opposite Strand/Antisense Transcript 1
*PEG3*: Paternally Expressed Gene 3
IVF: *In vitro* Fertilization
ICSI: Intracytoplasmic sperm injection
ROSI: Round spermatid injection
3Rs: Replacement, Reduction and Refinement
HTF: Human tubular fluid
PBS: Phosphate-buffered saline
RT: Room temperature
HBSS: Hanks' Balanced Salt Solution
RCF: Relative Centrifugal Force
NaCl: Sodium chloride
EDTA: Ethylenediamine tetraacetic acid
NaOH: Sodium hydroxide
SDS: Sodium dodecyl sulfate
DTT: DL-Dithiothreitol
rpm: Revolutions per minute
EtOH: Ethanol
TE: Tris EDTA buffer
RRBS: Reduced-representation bisulfite sequencing
PCR: Polymerase chain reaction
BSP: Bisulfite Sanger PCR sequencing
IPTG: Isopropyl β-d-1-thiogalactopyranoside
X-GAL: 5-Bromo-4-chloro-3-indolyl-β-d-galactopyranoside
NCBI: National Center for Biotechnology Information
*Tal2*: TAL BHLH Transcription Factor 2
*Sart3*: Spliceosome Associated Factor 3, U4/U6 Recycling Protein
*Oraov1*: LTO1 maturation factor of ABCE1
*Fam192*: Proteasome Activator Subunit 3 Interacting Protein 1
TBE: Tris-borate-EDTA
DMC: Differentially methylated CpGs
RT-qPCR: Reverse transcription quantitative real-time polymerase chain reaction
ANOVA: Analysis of variance
*Igf2*: Insulin-like growth factor 2
*Peg1*: Paternally expressed gene 1
