# Supplementary material for: Sperm DNA methylation is predominantly stable in mice offspring born after transplantation of long-term cultured spermatogonial stem cells
Source: Clin Epigenetics. 2023 Apr 7;15:58. doi: 10.1186/s13148-023-01469-x (PMC10080964; doi:10.1186/s13148-023-01469-x)
Supplement: Supplementary file 1 — Additional file 1. Supplementary tables and figures. [file 13148_2023_1469_MOESM1_ESM.docx]

**Supplementary data**

**Table S1**. DMRs of intergenic regions that displayed differential methylation spanning from F0 to F2, F2/M and F2/P, analyzed against datasets for known sequences of Intracisternal A-particle (IAP) sequences. F2/M – F2 generated from the maternal line, F2/P – F2 generated from the paternal line.

| Genomic location | Repeat chromosome | Repeat start | Repeat end | SSCT F0 + SSCT F1 + SSCT F2 | SSCT F0 + SSCT F1 + SSCT F2/M | SSCT F0 + SSCT F1 + SSCT F2/P | IAPs present |
| --- | --- | --- | --- | --- | --- | --- | --- |
| intergenic:4459 | chr3 | 91155001 | 91793078 | + | + | + | + |
| intergenic:6440 | **chr4** | **134969001** | **135033000** | **-** | **+** | **+** | **+** |
| intergenic:7407 | chr5 | 84968001 | 85648000 | - | - | + | + |
| intergenic:16036 | **chr11** | **9889001** | **9925000** | **-** | **-** | **+** | **-** |
| intergenic:19529 | chr13 | 61575001 | 61576000 | - | - | + | - |
| intergenic:24697 | **chr18** | **39977001** | **39978000** | **-** | **-** | **+** | **-** |

**Table S2**. Primers designed for BSP validation study of the RRBS resulting genes that are affected in multiple generations, and for RT-qPCR for gene expression analysis of *Tal2*.

| **Gene** | **Step** | **Forward primer sequence** | **Reverse primer sequence** |
| --- | --- | --- | --- |
| *Zfp229* | BSP | GGGAGTGATTAGGAGTGAATTTAT | TTAATCCCAACACTCAACAAACA |
| *Lefty* | BSP | AGGTTAGGAGGAGATATATATTAGGT | AAAAAATAAAATCCTTATAAAAAC |
| *Gpx8* | BSP | TTTAATTTTGTGGAAGATGGG | ATCTAAAAATATAAAATCTATCAAACTT |
| *Tal2* | BSP | GATTGGATTTTAGATGAAAGAATAA | CAATCCAATTCATAAATTAACAAAACTT |
| *F930017D23Rik* | BSP | TTATTAAGGAGGATTAGGGTGGT | CTTACTAATAAAATCAAAACTAAACTCCTA |
| *Nudt1* | BSP | TTATTGAGATAAGGAGTTGAGTA | CCACAAAATATCCACAAAATATC |
| *Oraov1* | RT-qPCR | TGATGGCGGATGAGAGATTCC | CCATGCAAGAGCAAAACCCC |
| *Fam192a* | RT-qPCR | TGCCACCTCCCTCCTTCATA | CGCCTCCTCTGGACATTCTTTT |
| *Sart3* | RT-qPCR | ATTACAACCTGGAACGGGCA | CACAGACGTGCTCAGGGTAG |
| *Tal2* | RT-qPCR | GCCTTTGCCAAGCTGAGGAAAC | AGGCTTTGCTCTCCCAAGACCT |

**Table S3.**  Validation by bisulfite Sanger sequencing at CpG level of the selected statistically significant hypo- and hyper-methylated genes found in several SSCT generations by RRBS.

|  |  | | RRBS | | | | | | | | | BSP | | | | | | | | | | |
| --- | --- | --- | --- | --- | --- | --- | --- | --- | --- | --- | --- | --- | --- | --- | --- | --- | --- | --- | --- | --- | --- | --- |
|  |  | | **F1 Control** | | | | **F1 SSCT** | | | | **F1 Control** | | | | | **F1 SSCT** | | | | | | |
| Gene | **CpG** | **Meth** | | **sd** | **Cov** | **sd** | **Meth** | **sd** | **Cov** | **sd** | **Meth** | | **sd** | **Cov** | **sd** | **Meth** | **sd** | | **Cov** | | **sd** | |
| *Gpx8* | 113045463 | 99 | | 0,03 | 10,6 | 3,5 | 67 | 0,57 | 12,67 | 10,02 | 94,42 | | 3,32 | 44,6 | 3,58 | 95,3 | 1,62 | | 44,4 | | 4,34 | |
|  | 113045507 | 100 | | 0 | 10,6 | 3,5 | 67 | 0,58 | 13 | 10,6 | 96,2 | | 4,35 | 45,4 | 3,51 | 97,78 | 2,32 | | 45,6 | | 5,08 | |
|  | 113045732 | 93 | | 0,17 | 50,2 | 24,3 | 68 | 0,27 | 78,4 | 48,4 |  | |  |  |  |  |  | |  | |  | |
|  | 113045734 |  | |  |  |  |  |  |  |  | 75,45 | | 11,30 | 57,20 | 9,28 | 72,18 | 5,41 | | 62,60 | | 4,28 | |
| *Zfp229* | 21730818 | 24 | | 0,1 | 27,4 | 9,9 | 0 | 0 | 31 | 12,8 | 2,44 | | 3,75 | 57,20 | 2,17 | 4,00 | 1,81 | | 54,80 | | 5,81 | |
| *Tal2* | 53786226/7 | 32,47 | | 21,17 | 59,4 | 32,94 | 3,02 | 4,20 | 35,80 | 9,20 | 23,42 | | 5,52 | 58,00 | 3,16 | 13,64 | 6,44 | | 57,60 | | 3,05 | |
|  | 53786542 | 15,88 | | 19,53 | 19,5 | 3,32 | 48,03 | 0 | 28,40 | 8,38 | 29,36 | | 6,84 | 57,60 | 2,51 | 31,88 | 15,96 | | 58,60 | | 1,52 | |
| *fRik* | 43623330/1 | 35,09 | | 29,94 | 71,2 | 37,27 | 2,25 | 3,24 | 54,80 | 24,08 | 15,10 | | 5,40 | 54,60 | 4,16 | 14,06 | 4,95 | | 56,20 | | 6,10 | |
|  | 43623585 | 4,65 | | 10,40 | 32,2 | 14,74 | 16,08 | 20,43 | 29,80 | 21,96 |  | |  |  |  |  |  | |  | |  | |
|  | 43623586 | 11,82 | | 16,22 | 46,2 | 33,67 | 10,64 | 14,66 | 43,00 | 16,55 | 5,08 | | 3,85 | 54,60 | 4,16 | 6,46 | 2,69 | | 56,20 | | 6,10 | |
| *Nudt1* | 140332760 |  | |  |  |  |  |  |  |  | 30,02 | | 12,17 | 54,60 | 2,41 | 39,52 | 6,61 | | 55,60 | | 3,13 | |
|  | 140332948 | 64,91 | | 44,95 | 31 | 16,36 | 92,61 | 16,53 | 35,60 |  |  | |  |  |  |  |  | |  | |  | |
|  | 140332950 |  | |  |  |  |  |  |  |  | 89,22 | | 7,81 | 54,60 | 2,41 | 87,94 | 4,77 | | 55,60 | | 3,13 | |
| *Lefty2* | 180893911 | 15,82 | | 9,43 | 47,2 | 5,97 | 29,70 | 39,47 | 23,50 | 11,79 | 42,26 | | 12,21 | 49,60 | 18,28 | 43,34 | 6,35 | | 59,60 | | 3,85 | |
|  |  | **F1 Control** | | | | | **F2 SSCT** | | | | **F1 Control** | | | | | **F2 SSCT** | | | | | | |
| Gene | CpG | Meth | | sd | Cov | sd | Meth | sd | Cov | sd | Meth | | sd | Cov | sd | Meth | | sd | | Cov | | sd |
| *Gpx8* | 113045463 | 99 | | 0,03 | 10,6 | 3,5 | 95 | 0,12 | 10,3 | 9,3 | 94,42 | | 3,32 | 44,6 | 3,58 | 97,1 | | 1,7 | | 53,4 | | 15,12 |
|  | 113045507 | 100 | | 0 | 10,6 | 3,5 | 96 | 0,12 | 10,25 | 8,6 | 96,2 | | 4,35 | 45,4 | 3,51 | 96,99 | | 2,52 | | 53,2 | | 13,85 |
|  | 113045732 | 93 | | 0,17 | 50,2 | 24,3 | 73 | 0,29 | 34 | 21,2 |  | |  |  |  |  | |  | |  | |  |
|  | 113045734 |  | |  |  |  |  |  |  |  | 75,45 | | 11,30 | 57,20 | 9,28 | 72,29 | | 6,16 | | 55,70 | | 4,97 |
| *Zfp229* | 21730818 | 24 | | 0,1 | 27,4 | 9,9 | 0 | 0 | 37,3 | 14,5 | 2,44 | | 3,75 | 57,20 | 2,17 | 3,27 | | 6,86 | | 57,70 | | 2,58 |
| *Tal2* | 53786226/7 | 32,47 | | 21,17 | 59,4 | 32,94 | 12,45 | 24,09 | 45,30 | 28,40 | 23,42 | | 5,52 | 58,00 | 3,16 | 15,30 | | 7,71 | | 58,90 | | 2,51 |
|  | 53786542 | 15,88 | | 19,53 | 19,5 | 3,32 | 18,62 | 33,77 | 21,50 | 9,06 | 29,36 | | 6,84 | 57,60 | 2,51 | 34,02 | | 8,99 | | 58,30 | | 1,77 |
| *fRik* | 43623330/1 | 35,09 | | 29,94 | 71,2 | 37,27 | 8,92 | 16,79 | 52,60 | 19,78 | 15,10 | | 5,40 | 54,60 | 4,16 | 14,88 | | 7,72 | | 55,30 | | 1,95 |
|  | 43623585 | 4,65 | | 10,40 | 32,2 | 14,74 | 0,80 | 2,53 | 24,70 | 8,23 |  | |  |  |  |  | |  | |  | |  |
|  | 43623586 | 11,82 | | 16,22 | 46,2 | 33,67 | 12,69 | 16,18 | 35,40 | 14,83 | 5,08 | | 3,85 | 54,60 | 4,16 | 6,51 | | 1,89 | | 55,30 | | 1,95 |
| *Nudt1* | 140332948 | 64,91 | | 44,95 | 31,0 | 16,36 | 78,53 | 18,48 | 39,33 | 20,18 |  | |  |  |  |  | |  | |  | |  |
|  | 140332950 |  | |  |  |  |  |  |  |  | 89,22 | | 7,81 | 54,60 | 2,41 | 86,27 | | 4,02 | | 58,35 | | 12,98 |
| *Lefty2* | 180893911 | 15,82 | | 9,43 | 47,2 | 5,97 | 54,94 | 23,99 | 28,20 | 13,93 | 42,26 | | 12,21 | 49,60 | 18,28 | 37,97 | | 6,51 | | 56,20 | | 3,79 |
|  |  | **F1 Control** | | | | | **F2/M** | | | | **F1 Control** | | | | | **F2/M** | | | | | | |
| Gene | CpG | Meth | | sd | Cov | sd | Meth | sd | Cov | sd | Meth | | sd | Cov | sd | Meth | | sd | | Cov | | sd |
| *Gpx8* | 113045463 | 99 | | 0,03 | 10,6 | 3,5 | 89 | 0,19 | 9 | 8,19 | 94,42 | | 3,32 | 44,6 | 3,58 | 97,62 | | 1,71 | | 58,8 | | 20,9 |
|  | 113045507 | 100 | | 0 | 10,6 | 3,5 | 89 | 0,19 | 9 | 8,2 | 96,2 | | 4,35 | 45,4 | 3,51 | 96,78 | | 3,04 | | 58 | | 19,18 |
|  | 113045732 | 93 | | 0,17 | 50,2 | 24,3 | 62 | 0,35 | 34 | 17,3 |  | |  |  |  |  | |  | |  | |  |
|  | 113045734 |  | |  |  |  |  |  |  |  | 75,45 | | 11,30 | 57,20 | 9,28 | 73,98 | | 5,79 | | 57,40 | | 2,41 |
| *Zfp229* | 21730818 | 24 | | 0,1 | 27,4 | 9,9 | 0 | 0 | 34,2 | 13,8 | 2,44 | | 3,75 | 57,20 | 2,17 | 5,80 | | 9,43 | | 59,20 | | 0,84 |
| *Tal2* | 53786068 |  | |  |  |  |  |  |  |  | 19,34 | | 7,56 | 58,00 | 3,16 | 8,92 | | 3,99 | | 58,60 | | 2,70 |
|  | 53786110 |  | |  |  |  |  |  |  |  | 20,70 | | 6,76 | 58,00 | 3,16 | 10,32 | | 3,87 | | 58,60 | | 2,70 |
|  | 53786136 |  | |  |  |  |  |  |  |  | 20,46 | | 4,29 | 57,80 | 3,11 | 12,84 | | 6,80 | | 58,60 | | 2,70 |
|  | 53786196 |  | |  |  |  |  |  |  |  | 23,60 | | 3,26 | 58,00 | 3,16 | 11,40 | | 4,96 | | 58,60 | | 2,70 |
|  | 53786214 |  | |  |  |  |  |  |  |  | 26,36 | | 4,06 | 58,00 | 3,16 | 15,50 | | 5,74 | | 58,60 | | 2,70 |
|  | 53786226/7 | 32,47 | | 21,17 | 59,4 | 32,94 | 14,84 | 33,18 | 53,60 | 33,73 |  | |  |  |  |  | |  | |  | |  |
|  | 53786288 |  | |  |  |  |  |  |  |  | 21,85 | | 3,16 | 58,00 | 3,16 | 12,22 | | 5,24 | | 58,60 | | 2,70 |
|  | 53786542 | 15,88 | | 19,53 | 19,5 | 3,32 | 17,23 | 23,69 | 22,00 | 8,97 | 29,36 | | 6,84 | 57,60 | 2,51 | 35,40 | | 7,97 | | 58,00 | | 2,35 |
| *fRik* | 43623585 | 4,65 | | 10,40 | 32,2 | 14,74 | 0,00 | 0,00 | 26,60 | 7,92 |  | |  |  |  |  | |  | |  | |  |
|  | 43623586 | 11,82 | | 16,22 | 46,2 | 33,67 | 14,13 | 14,97 | 38,40 | 18,11 | 5,08 | | 3,85 | 54,60 | 4,16 | 6,74 | | 2,65 | | 56,40 | | 0,55 |
| *Nudt1* | 140332750 |  | |  |  |  |  |  |  |  | 16,56 | | 5,48 | 54,60 | 2,41 | 14,14 | | 6,16 | | 48,32 | | 17,82 |
|  | 140332948 | 64,91 | | 44,95 | 31 | 16,36 | 72,1 | 16,08 | 36,00 | 19,08 |  | |  |  |  |  | |  | |  | |  |
|  | 140332950 |  | |  |  |  |  |  |  |  | 89,22 | | 7,81 | 54,60 | 2,41 | 85,10 | | 5,00 | | 62,90 | | 17,24 |
| *Lefty2* | 180893911 | 15,82 | | 9,43 | 47,2 | 5,97 | 52,1 | 33,15 | 31,80 | 15,85 | 42,26 | | 12,21 | 49,60 | 18,28 | 40,74 | | 5,53 | | 55,60 | | 4,83 |
|  | 180893912 |  | |  |  |  |  |  |  |  | 42,26 | | 12,21 | 49,60 | 18,28 | 40,74 | | 5,53 | | 55,60 | | 4,83 |
|  |  | **F1 Control** | | | | | **F2/P** | | | | **F1 Control** | | | | | **F2/P** | | | | | | |
| Gene | CpG | Meth | | sd | Cov | sd | Meth | sd | Cov | sd | Meth | | sd | Cov | sd | Meth | | sd | | Cov | | sd |
| *Gpx8* | 113045463 | 99 | | 0,03 | 10,6 | 3,5 | 99 | 0,02 | 11,25 | 11,2 | 94,42 | | 3,32 | 44,6 | 3,58 | 96,4 | | 1,62 | | 48 | | 2,24 |
|  | 113045507 | 100 | | 0 | 10,6 | 3,5 | 100 | 0 | 11,25 | 11,2 | 96,2 | | 4,35 | 45,4 | 3,51 | 97,2 | | 2,22 | | 48,4 | | 2,51 |
|  | 113045509 |  | |  |  |  |  |  |  |  | 93,56 | | 5,24 | 57,20 | 9,28 | 93,32 | | 0,63 | | 54,00 | | 6,52 |
|  | 113045732 | 93 | | 0,17 | 50,2 | 24,3 | 83 | 0,19 | 34 | 24,8 |  | |  |  |  |  | |  | |  | |  |
|  | 113045734 |  | |  |  |  |  |  |  |  | 75,45 | | 11,30 | 57,20 | 9,28 | 70,60 | | 6,70 | | 54,00 | | 6,52 |
| *Zfp229* | 21730817/8 | 24 | | 0,1 | 27,4 | 9,9 | 0 | 0 | 40 | 16,1 | 2,44 | | 3,75 | 57,20 | 2,17 | 0,74 | | 1,01 | | 56,20 | | 2,95 |
| *Tal2* | 53786226/7 | 32,47 | | 21,17 | 59,4 | 32,94 | 10,06 | 13,8 | 37 | 22,46 | 23,42 | | 5,52 | 58,00 | 3,16 | 18,46 | | 8,54 | | 59,20 | | 2,59 |
|  | 53786542 | 15,88 | | 19,53 | 19,5 | 3,32 | 20 | 44,72 | 21,00 | 10,17 | 29,36 | | 6,84 | 57,60 | 2,51 | 31,88 | | 15,96 | | 58,60 | | 1,52 |
| *fRik* | 43623330/1 | 35,09 | | 29,94 | 71,2 | 37,27 | 2,12 | 4,74 | 60,60 | 21,22 |  | |  |  |  |  | |  | |  | |  |
|  | 43623585 | 4,65 | | 10,40 | 32,2 | 14,74 | 1,6 | 3,58 | 22,80 | 18,65 |  | |  |  |  |  | |  | |  | |  |
|  | 43623586 | 11,82 | | 16,22 | 46,2 | 33,67 | 11,25 | 18,96 | 32,40 | 12,03 | 5,08 | | 3,85 | 54,60 | 4,16 | 6,28 | | 0,92 | | 54,20 | | 2,28 |
| *Nudt1* | 140332948 | 64,91 | | 44,95 | 31 | 16,36 | 84,97 | 21,20 | 47,00 | 21,92 |  | |  |  |  |  | |  | |  | |  |
|  | 140332950 |  | |  |  |  |  |  |  |  | 89,22 | | 7,81 | 54,60 | 2,41 | 87,44 | | 2,83 | | 53,80 | | 5,50 |
| *Lefty2* | 180893911 | 15,82 | | 9,43 | 47,2 | 5,97 | 57,77 | 0,13 | 24,60 | 12,36 | 42,26 | | 12,21 | 49,60 | 18,28 | 35,20 | | 6,75 | | 56,80 | | 2,86 |

**
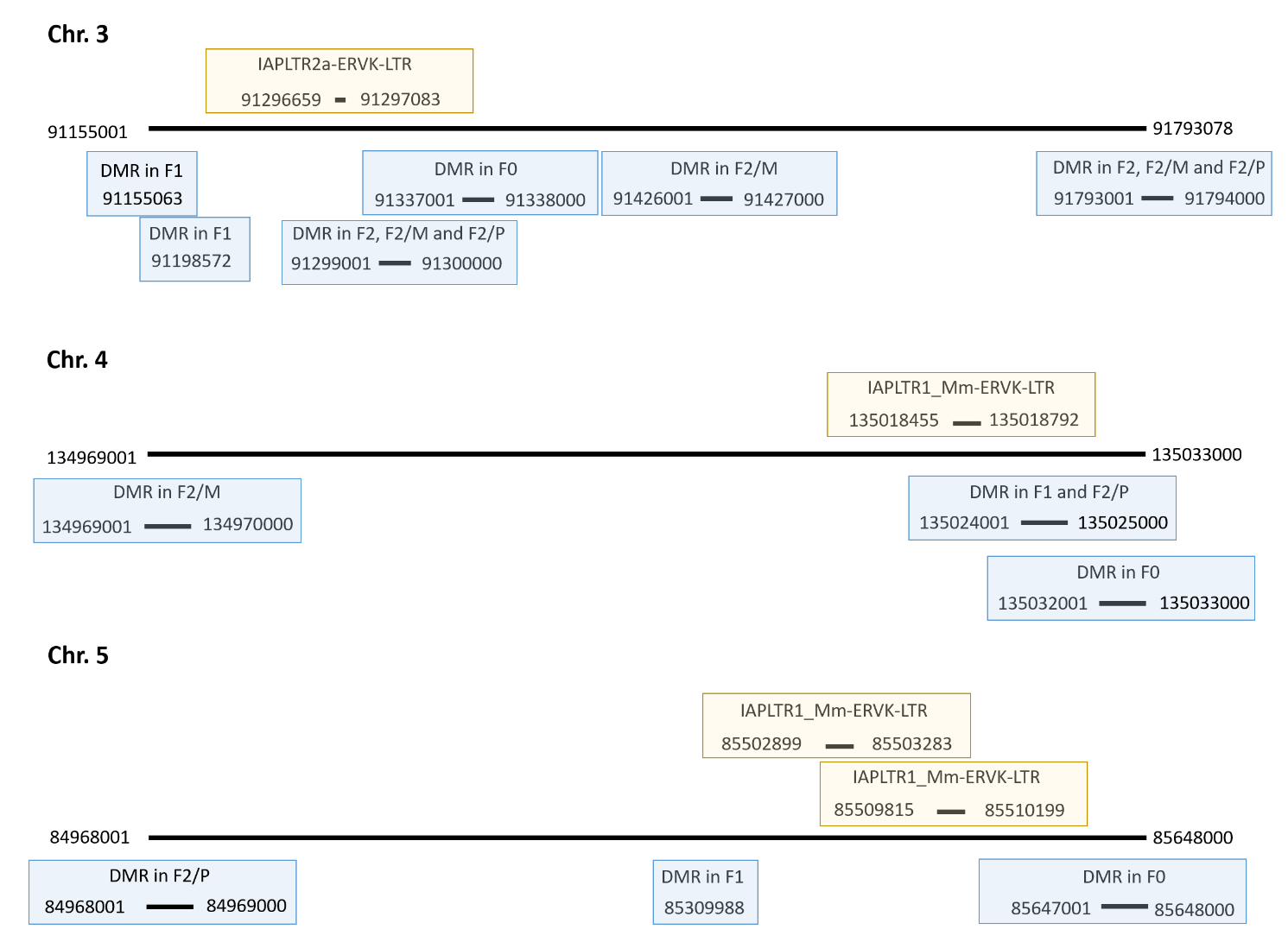
 Figure S1.** Genomic intergenic regions on chromosome 3, 4 and 5 showing annotation of the DMRs that displayed differential methylation spanning from F0 to F2, F2/M and F2/P. F2/M – F2 generated from the maternal line, F2/P – F2 generated from the paternal line compared to the annotation of existing IAPs in the same intergenic region.

**
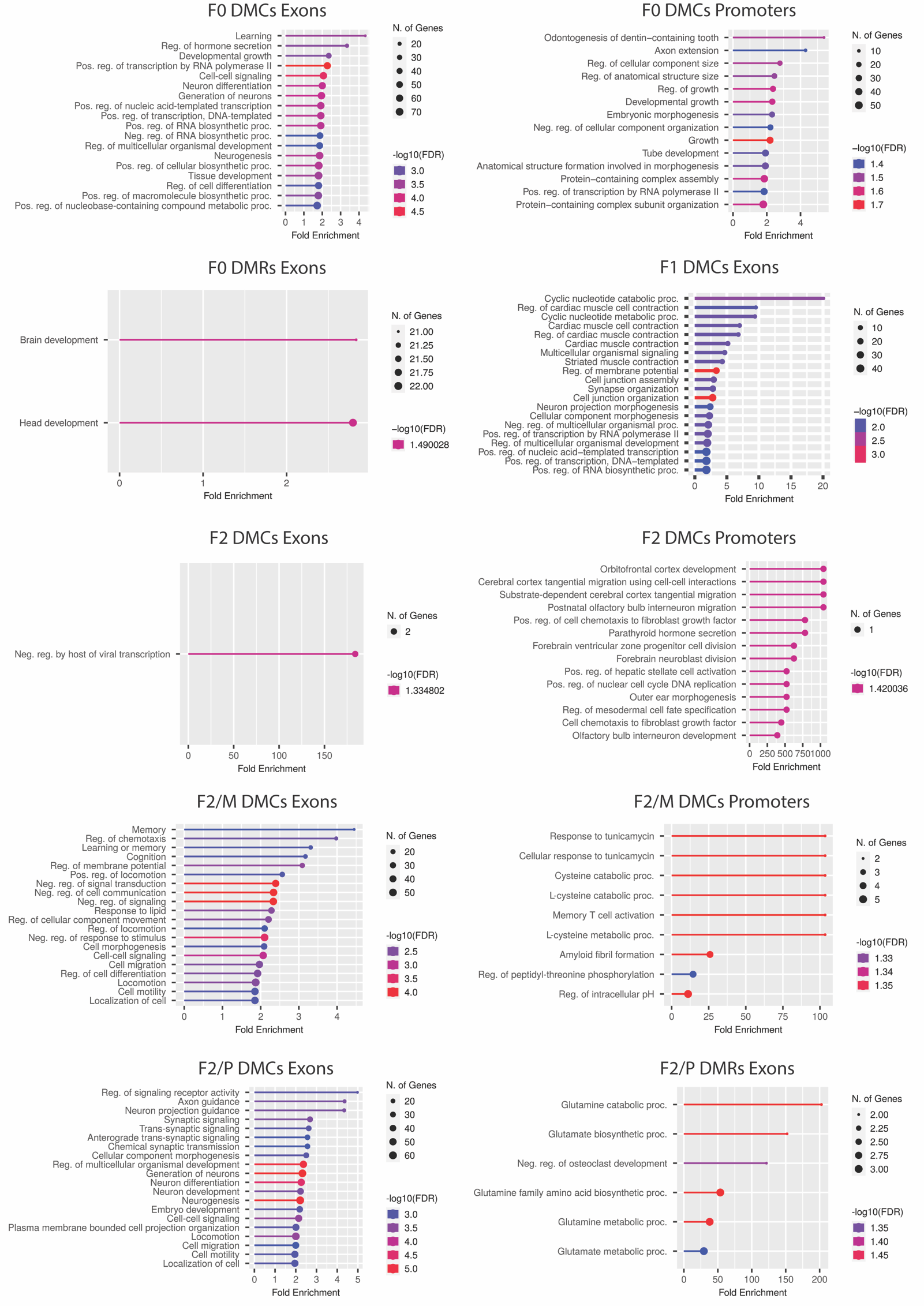
**

**Figure S2.** Gene ontology analysis, biological association of genes annotated to DMRs in exon and promoter regions in F1, F2 SSCT/M or F2 SSCT/M versus control.
